# Dynamic Fusional Vergence Eye Movements in Congenital Esotropia

**DOI:** 10.2174/1874364100802010009

**Published:** 2008-02-06

**Authors:** Yair Morad, Horace Lee, Carol Westall, Stephen P Kraft, Carole Panton, Ruth Sapir-Pichhadze, Moshe Eizenman

**Affiliations:** 1 Department of Ophthalmology, The Hospital for Sick Children, and the University of Toronto, Toronto, Ontario, Canada; 2 Institute of Biomedical Engineering, University of Toronto, Toronto, Ontario, Canada

**Keywords:** Strabismus, infantile esotropia, vergence, fusional disparity.

## Abstract

**Purpose:**

To evaluate whether a selected group of 9 children with history of congenital esotropia is capable of producing vergence eye responses to fusional disparity stimuli.

**Methods:**

Nine children with history of congenital esotropia and 5 age-matched children with normal binocular vision were examined. Using a full-field target, vergence responses to base out 3 prism diopters placed in front of both eyes were recorded.

**Results:**

In five patients, the initial response was a saccade generated by the dominant eye, followed by a disconjugate movement of one or both eyes. In two patients with long standing uncorrected strabismus, the responses were almost purely saccadic, while in two other patients, in whom early surgery resulted in fusional abilities, smooth vergence movements were recorded.

**Conclusion:**

This study adds further evidence that patients with history of congenital esotropia patients are capable of producing vergence eye movements in response to fusional disparity. The responses usually start with a saccade followed by a vergence response. The preference for initial saccadic or vergence response is correlated with sensorial tests of stereopsis and motor fusion and may be related to the size of the suppression scotoma in the deviating eye, the duration of misalignment, or both.

Congenital esotropia is one of the most common forms of strabismus, affecting 0.5-1% of all children [1]. Nevertheless, the pathogenesis of this common disorder is still poorly understood. Over the years, theories have developed as to the etiology of congenital esotropia; Worth's sensory theory maintained that congenital esotropia results from a congenital, irreparable sensory defect in the visual cortex that prevents normal alignment of the eyes. Chavasse’s motor theory, on the other hand, maintained that children with congenital esotropia have the capacity for good binocular vision, however, mal-development of the motor reflexes or deficits in the projections of the binocular sensory information to the oculomotor system is the cause for eye deviation and misalignment [2]. The characteristics of vergence eye-movements in patients with congenital esotropia strabismus, and their relationship to the disease and its treatment have long been a matter of debate. Based on subjective measurements, Burian demonstrated that such eye-movements exist when the peripheral visual field is stimulated [3], even in patients with abnormal retinal correspondence. Parks disagreed with this observation and suggested that patients with abnormal retinal correspondence cannot have fusional vergence movements [4]. Kenyon *et al.*, using binocular eye-movements recordings, maintained that vergence eye-movements in strabismic patients are limited to accommodative vergence, while fusional vergence is absent [5, 6]. Boman and Kertesz, on the other hand, successfully demonstrated vergence responses to fusional disparity stimuli in patients with congenital esotropia. However, these responses were suboptimal [7].

In view of these previous findings, the purpose of this study was to evaluate vergence responses in patients with history of congenital esotropia without amblyopia, in order to ascertain whether these children are capable of eliciting normal or near-normal responses to fusion disparity stimuli.

## METHODS

The study group included 9 children with history of congenital esotropia and 5 children with normal binocular vision. The study was approved by the Ethics Review Board of the Hospital for Sick Children, Toronto, Canada. All study and control patients underwent complete ocular and orthoptic examination. Inclusion criteria for the study patients were: esotropia diagnosed before 6 months of age that could not be corrected with glasses; refraction of less than +2.0 diopters; visual acuity (VA) of 6/12 or better in each eye; a difference of up to one Snellen line in the visual acuity between the two eyes; absence of any ocular pathology apart from strabismus; and absence of any neurological abnormality such as cerebral palsy. Control subjects had normal binocular vision and normal ophthalmological examination. All subjects were fitted with an infrared binocular eye tracker, (El-MAR series 2000 Binocular Eye Tracker, Toronto, Ontario, Canada) that measured the subjects’ eye positions at a sampling rate of 120 Hz. This particular eye tracker allows patients to wear their glasses while recording the eye-movements. Subjects were seated 100 cm in front of a target with their heads supported by a chin rest. All subjects wore their prescribed optical correction. Calibration was done separately for each eye. The visual stimulus was a black cross on a white background (71cmX56cm - 39˚ horizontal, 31˚ vertical). A circular 1'' diameter picture of an orange sun placed in the center of the cross was used as a fixation point. Three diopter Fresnel prisms were mounted onto a hinged frame that was attached to the eye-tracker. By moving the hinged frame up or down, the three prism diopters were simultaneously introduced into each eye’s field of view for 10-15 seconds and then removed from the subject’s field of view for 10-15 seconds. The prisms were presented to both eyes in a base-out configuration (i.e. to introduce crossed disparities). This procedure was repeated 10 times. Subjects were told that if they see the target double when the prisms were introduced into their field of view, they should try to fuse the images from both eyes and maintain clear view of the fine details of the orange sun (on which a smiling face was drown).

Data was analyzed off-line, by semi-automated algorithms that classified and quantified the eye-movement responses. The instantaneous horizontal vergence angle was calculated by subtracting the horizontal position of the left eye from that of the right eye. The conjugate response was calculated as the average of the horizontal positions of the right and left eyes.

## RESULTS

The mean age of patients with history of congenital esotropia and children with normal binocular vision was 9.38±0.9 and 10.23±0.5 years respectively (*P=*0.78). Detailed information regarding the clinical profile of the strabismus patients is presented in the Table **1**. All the children with normal binocular vision had VA of 6/9 or better in each eye, with orthophoria on cover-uncover and alternate cover tests. They all fused on Worth 4-Dot for distance and near, had normal stereoacuity on the Random Dot test and the Fly test and normal retinal correspondence in the Bagolini test.

Typical eye-movement responses of a child with normal binocular vision to the introduction of base out 3-prism diopters Fresnel prisms is shown in Fig. (**1A**). The left panel of Fig. (**1A** (L)) shows the eye-movement responses of the left and right eyes. Both eyes moved in a smooth disconjugate manner in response to the introduction of the prisms (i.e. normal vergence response). The middle panel (M) shows the disconjugate component of the binocular eye-movements and the right panel (R) shows the conjugate component of the binocular eye-movements. The vergence amplitude (M) was approximately 3 degrees (the expected response to 3 prism diopters to each eye) and the conjugate component (R) was approximately zero. All children with normal binocular vision responded similarly to all fusional disparity stimuli.

Eye-movements in response to fusional disparity stimuli in patients with history of congenital esotropia showed greater variability. Most patients with history of congenital esotropia were unable to generate smooth disconjugate eye-movements. Typical responses were composed of conjugate, often asymmetric, saccadic responses and disconjugate, often monocular, slow drifts. The end result of the combined sequence of movements only partially compensated for the crossed disparity introduced by the visual stimuli. Typical eye movement recordings from patient 4 following the introduction of 3 prism diopters base out prisms in front of each eye is presented in Fig. (**1B**). The initial eye movement was a vergence response (started at approximately 24.2 seconds see Fig. (**1B**) Middle) with duration of 250msec followed by a 1 degree conjugate saccade and then a monocular drift of the non-dominant left eye. The total vergence amplitude (2 degrees) was less than the amplitude of the responses recorded in subjects with normal binocular vision. Another typical response is presented in Fig. (**1C**) (patient 3). The response started with a conjugate saccade, which served to foveate the target with the dominant right eye. Following that, the left eye performed a smooth eye movement (drift) in a direction that was opposite to that of the initial saccade. This pattern was also recorded in patient 1,2, and 5 (data not shown). 5/9 patients exhibited this combined pattern of saccade-vergence responses.

**Fig. (2):**
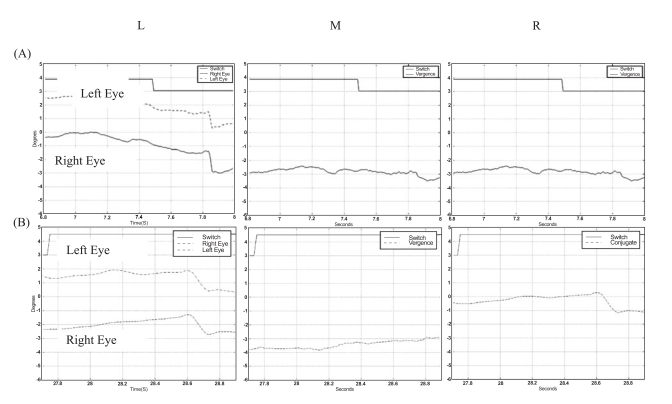
^*^ Left LTypical eye-movement responses of patients 9 **(A)** and 6 **(B)** who had prolonged un-corrected strabismus. Both patients responded with saccadic eye-movements with little or no vergence. Middle (M): Vergence response, (right eye - left eye). Right (R): Conjugate response, (right eye + left eye)/2. ^*^ In the figure movement of the eye to the right is displayed downward, and movement to the left is displayed upward in the graph.

In Fig. (**2A**) eye-movements recordings from patient 9 are presented. This patient had undergone 6 strabismus surgeries, and demonstrated complete suppression of the left eye. In this patient, only a very limited disconjugate (vergence) response was apparent (see Fig. (**2A**) (M)). The dominant feature in his responses was a conjugate saccadic response in which the dominant left eye foveated the target. Similar responses were generated by patient 6 who also had complete suppression of the non-dominant eye (Fig. **2B**). This patient had undergone the first surgery at the age of 3 years, whereas the other patients in this study had their first surgery between 8-18 months of age.

**Fig. (3):**
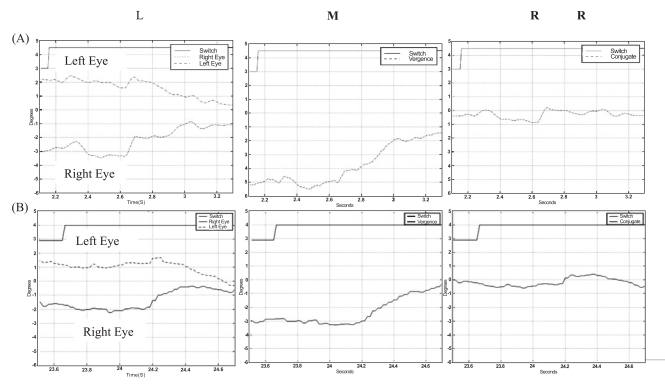
^*^Left L Typical eye-movement responses of patients 7 **(A)** and 8 **(B)** which demonstrated some binocular fusion capacity. Middle (M): Vergence response, (right eye-left eye). Right (R): Conjugate response, (right eye+left eye)/2.

^*^ In the figure movement of the eye to the right is displayed downward, and movement to the left is displayed upward in the graph.

Patients 7 and 8 were the only patients in the study who could fuse the Worth 4-Dot target at 1 meter and had a positive response on the Fly test. They also displayed a positive response on the Fly Test. Their responses to fusional disparity vergence stimuli consisted of a saccade followed by a smooth vergence movement (see Fig. **3A**) or smooth vergence movements with a very limited conjugate saccadic components (see Fig. **3B**).

## DISCUSSION

Normal fusional vergence eye-movements have been well documented in the literature [8, 9]. The smooth disconjugate eye-movements recorded in subjects with normal binocular vision serve to overcome crossed disparity and to foveate the target with the two eyes. Although such smooth vergence responses were almost never recorded in our subjects with history of congenital esotropia, most of our patients were capable of generating compensatory disconjugate eye-movements to fusional disparity vergence stimuli. Different patients had a different strategy of generating the disconjugate responses; In most patients, an initial saccadic movement driven by the dominant eye served to foveate the it on the target. This was followed by a smooth disconjugate drift of the non-dominant eye in a direction that compensated for the disparity introduced by the stimuli. Similar observations were reported by Kenyon *et al*. [6] in a study of accommodative vergence in strabismic patients. A later study by Boman and Kertesz also found that an early binocular saccadic response was used to foveate the target with the dominant eye. This early response was followed by binocular accommodative vergence movements [7]. Kenyon noted that during the disconjugate movements, the amplitude of the dominant eye was always smaller than that of the non-dominant eye. Our patients demonstrated a similar response to fusional disparity stimuli. Kenyon also postulated that the presence of a suppression scotoma in the non-dominant eye could lead to abnormal disparity vergence responses in these patients.

The responses of patients 6 and 9 to fusional disparity vergence stimuli consisted almost entirely of conjugate eye-movements. These patients were clinically different from the rest of the patients because of their surgical history: patient 6 was first operated on at the age of three years. He demonstrated total suppression of the right eye in all tests. Patient 9 had undergone 6 strabismus surgeries prior to the examination. This patient had had long periods of non-alignment that might have resulted in the development of a large suppression scotoma in the right eye. Patients 7 and 8 were the only subjects who could generate smooth disconjugate eye-movements (i.e. devoid of saccades). These patients were distinct from the other subjects by their ability to fuse on Worth 4-Dot test at one meter, a distance equal to the distance of the visual stimulus, and by displaying gross stereoacuity on the Fly Test. Both were operated on at a relatively early age of 8 months and had a small residual deviation of 2-6 diopters. This observation is supported by a study by Tychsen *et al*. in macaque monkeys. in which a monkey with an infantile large angle esotropia had almost no convergence movements, while another monkey with small angle esotropia achieved much better responses, although asymmetric with subnormal vergence amplitudes [10]. It is well established that the binocular capabilities of patients with history of congenital esotropia who had surgery after 2 years of age are likely to be inferior to the post operative results of patients operated at an earlier age [11]. Early operation leads to better stereoacuity results and better alignment [12, 13]. Birch *et al*. reported that congenital esotropia patients with better stereoacuity have a better chance of maintaining good alignment [14]. An explanation for the difference between patients who had long periods of misalignment and patients who had relatively short periods of misalignment was suggested by Tychsen and Wong [15]. They showed that strabismus that was artificially created at a young age in monkeys had a disruptive effect on the development of binocular connections in area V1 of the cortex. This effect was correlated with the duration of misalignment, and could be halted by early correction of ocular alignment. It is possible that early surgery provides an opportunity for binocular connections to develop in area V1. This may improve the patients’ capacity for sensory fusion and thus enhance the sensory input to the vergence control system. This may improve the ability of the vergence control system to keep the eyes aligned. Better ocular alignment will in turn improve the probability that binocular connections in area V1 will further develop and thus further improve vergence capabilities. This positive feedback cycle may explain the improved vergence response in patients 7 and 8.

Another possible explanation for the large differences in responses to fusional disparity vergence stimuli among our patients may be related to the size of suppression scotoma in the non-dominant eye. It is well established that patients with congenital esotropia develop suppression scotomas in the deviating eye in order to eliminate diplopia. The size of this scotoma is inversely related to the angle of deviation. Patients 7 and 8 had relatively small suppression scotomas, as evident by their ability to fuse the Worth 4 Dot. Patients 6 and 9 on the other hand, had large suppression scotomas that developed over the long period of uncorrected strabismus. It is possible that in children with history of congenital esotropia the central portion of the target that is seen monocularly (due to the presence of a suppression scotoma) acts as a stimulus for saccadic eye-movements. At the same time, the portion of the target that is seen peripherally by both eyes acts as a stimulus for vergence eye-movements. The decision to generate either a saccadic or vergence response is therefore related to the relative magnitude of the saccadic and vergence stimuli and therefore to the diameter of the suppression scotoma. Adults with normal binocular vision who viewed vergence stimuli with artificial monocular scotomas generated “sub-optimal” vergence eye-movements that were similar to those observed in children with history of congenital esotropia [16]. As the size of the artificial scotoma increased, the capacity to generate smooth vergence eye-movements decreased [17], and the probability of generating optimal vergence responses was reduced. Boman and Kertesz [7] showed that the overall motor vergence response in subjects with normal binocular vision was deficient when a central scotoma was superimposed on images presented either to one of the eyes or both eyes. This deficient response was demonstrated only for scotomas larger than 5°. Smaller scotomas did not impede the vergence response. Although not specifically referred to in the text, it can be appreciated from the eye movement recordings presented in the paper, that vergence responses to stimuli containing monocular artificial scotomas had saccadic components similar to the those we found in our patients.

In conclusion, the results of this study suggest that children with history of congenital esotropia can generate vergence eye-movements in response to fusional disparity stimuli. The responses are variable and composed of both saccadic and vergence components. The preference for initial saccadic or vergence responses may be related to the size of the suppression scotoma in the deviating eye, the duration of misalignment, or both.

## Figures and Tables

**Fig. (1):**
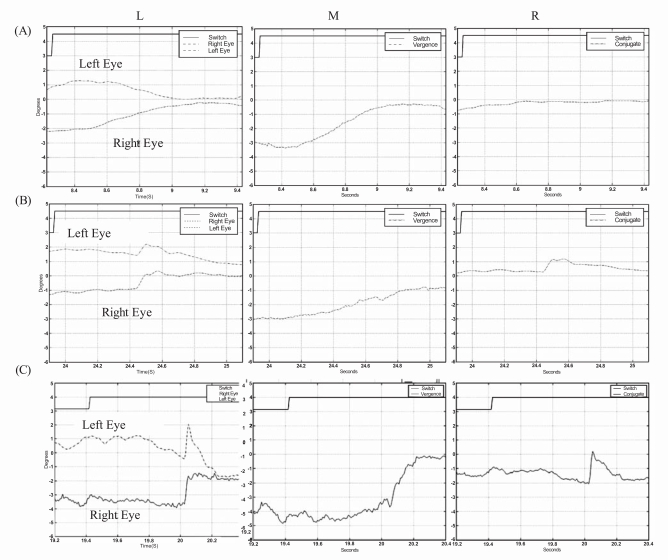
^*^(A). Typical eye-movement responses of a child with normal binocular vision to the introduction of 3 prism diopter base-out prisms to each eye. ** (B,C)**: Examples of typical eye movement responses of patients with history of congenital esotropia to fusional disparity vergence stimuli. Horizontal eye-movements of the left and right eyes are shown in plate (L) column. A positive change in eye position represents movement to the right. Vergence eye-movements (M): Vergence response, (right eye - left eye). Positive changes in vergence amplitude represent convergence. Version eye-movements (R): Conjugate response, (right eye + left eye)/2. In each image, the switch signal represents the introduction of the prisms into the subjects’ field of view. ^*^In the figure movement of the eye to the right is displayed downward, and movement to the left is displayed upward in the graph.

**Table 1.** Patient’s Clinical and Demographic Data

Patient no, Age, Sex	Age at First Surgery/ Number of Surgeries	Visual Acuity	Correction	Deviation in Prism Diopters (1 Meter/6 Meters)	Correspondence with Bagolini Test	Worth 4-Dot’s Test (1 meter/6 Meters)	Stereoacuity
1/11/F	18 mo./2	R: 6/12 L: 6/12^-1^	R: +1.00 L: +2.00	LET 10/LET 4	ARC	LE supp/LE Supp	RanDot=0 Fly=0
2./8/F	16 mo./1	R: 6/7.5 L:6/6	None	RET 14/RET 12	ARC	RE supp/RE supp	RanDot=0 Fly=0
3/10/M	13 mo./2	R:6/6 L:6/9	None	LET 16/LET 14	LE supp	LE supp/LE supp	RanDot=0 Fly=0
4/8/M	14 mo./1	R: 6/9 L: 6/12^+2^	R: +1.00 L: +1.50	LET 4/LET 4	ARC	LE supp/LE supp	RanDot=0 Fly=0
5/9/F	14 mo./2	R: 6/6 L: 6/6	None	RXT 8/RXT 14	RE supp	RE supp/RE supp	RanDot=0 Fly=0
6/10/M	36 mo./2	R: 6/9^+2^ L: 6/9^+2^	None	RXT 6/RXT 6	ARC	RE supp/RE supp	RanDot=0 Fly=0
7/10/M	8 mo./2	R: 6/6^-2^ L: 6/6^-3^	None	LET 6/LET 6	ARC	Fusion/LE supp	RanDot=0 Fly positive
8/10/M	8 mo./3	R: 6/6 L: 6/6^-2^	None	LXT 2/LXT 4	ARC	Fusion/Fusion	RonDot=0 Fly positive
9/9/M	18 mo./6	R: 6/6^-2^ L: 6/7.5^-2^	None	LXT 10/10 LXT	LE supp	LE supp/LE supp	RanDot=0 Fly=0

ARC- abnormal retinal correspondence, Supp- suppression, RE-right eye, LE- Left eye, RET/LET – right/left esotropia, RXT/LXT- right/left exotropia. RandDot – Random Dot E stereoacuity test, Fly – Titmus fly test.
